# Plasticity in leader–follower roles in human teams

**DOI:** 10.1038/s41598-017-14851-6

**Published:** 2017-11-06

**Authors:** Shinnosuke Nakayama, Manuel Ruiz Marín, Maximo Camacho, Maurizio Porfiri

**Affiliations:** 1 Department of Mechanical and Aerospace Engineering, New York University Tandon School of Engineering, 6 Metrotech Center, Brooklyn, NY 11201 USA; 2 Department of Quantitative and Informatic Methods, Technical University of Cartagena, Plaza Cronista Isidoro Valverde, 30202 Cartagena, Spain; 3 Department of Quantitative Methods for Economics and Business, University of Murcia, Campus de Espinardo, Murcia, 30100 Spain

## Abstract

In humans, emergence of leaders and followers is key to group performance, but little is known about the whys and hows of leadership. A particularly elusive question entails behavioral plasticity in leadership across social contexts. Addressing this question requires to eliminate social feedback between focal individuals and their partners in experiments that could illuminate the spontaneous emergence of social roles. We investigated plasticity in leader–follower roles in cooperation, where members choose the task toward a shared goal, and coordination, where members adjust their actions in real time based on social responsiveness. Through a computer-programmed virtual partner, we demonstrate adaptive plasticity in leader–follower roles. Humans increased their followership to cooperate when the partner led more in the choice of the task, whereas they showed only weak leadership when the partner followed more. We leveraged the information-theoretic notion of transfer entropy to quantify leadership and followership in coordination from their movements. When exhibiting stronger followership in task cooperation, humans coordinated more with the partner’s movement, with greater information being transferred from the partner to humans. The evidence of behavioral plasticity suggests that humans are capable of adapting their leader–follower roles to their social environments, in both cooperation and coordination.

## Introduction

From small teams to giant corporations, emergence of leaders and followers is key to enhancing group performance^1^. Leadership is often found to be highly consistent across contexts^2,3^, possibly because of its link to personality traits, such as initiative, assertiveness, and extraversion^4–6^. Leadership could also be situational, whereby some studies show that leadership traits depend on the group task^7–9^. Far from clear is our understanding of how interrelationships in a social environment shape leadership: we know little on whether and how humans adapt their roles of leader and follower in response to their social partners. Such an adaptation may, in turn, influence the overall group performance, resulting more or less effective human teams working toward a shared goal.

Contrary to our intuition, behavioral plasticity in unpredictable social environments has been proposed to be evolutionarily unfavorable^10–12^. However, it may be tenable to hypothesize that behavioral plasticity could be favored over consistency if the benefits exceeded the costs, such as collecting relevant information and adaptively adjusting the choice of actions^13^. Indeed, in a zero-sum game that requires cooperation in the choice of tasks, the optimal solution is achieved by the emergence of consistent leadership and followership^14^. When one has a strong expectation for others’ choices, however, the optimal solution is to adopt a maximin strategy, that is, players maximize the minimum payoff by switching the leader–follower roles based on the expectation^14^. These insights suggest that expectation for others’ choice is key to understanding leader–follower roles in task cooperation toward a shared goal.

Another aspect of leadership and followership is found in the coordination between group members in real time based on social responsiveness, which could be expressed differently from those in task cooperation. The information-theoretic construct of transfer entropy is a promising tool to quantify the extent of social responsiveness, thereby offering an unbiased, model-free perspective to study leader–follower relationship in coordination^15^. Specifically, transfer entropy measures the reduction of the uncertainty in predicting the future state of one process, given the current state of the other process^16^. This concept has been applied to unveil causality between processes across a number of research domains, such as neuroscience^17^, molecular biology^18^, social media^19^, and climate networks^20^. In collective behavior of animals, transfer entropy has been successfully used to identify leader–follower relationship during coordinated movements^21–23^. Therefore, an information-theoretic analysis promises to disentangle and quantify social interactions in coordinating humans.

In contrast to a rich literature on leadership traits and processes, empirical studies on plasticity in leader–follower roles under different social environments are limited^1^, possibly due to the difficulty in controlling the systems. The use of a computer-programmed virtual agent offers a unique opportunity to enhance system controllability, affording to elucidate the spontaneous emergence of leader–follower roles. Social exchange theory posits that leadership and followership emerge through a feedback loop between individuals^24^. That is, the behavior of an individual is not the direct response to their partners, but the synergistic outcome of a social feedback loop. To pinpoint the direct behavioral response to social partners, it is important to eliminate the social feedback between focal individuals and their social partners. Through a computer-programmed virtual partner, one can identify the underlying leadership and followership processes by excluding social feedback in collaborating groups, thereby controlling for the partner’s propensity from changing.

We examined whether humans adaptively change their leader–follower roles in task cooperation and movement coordination when working with a virtual partner that has a different propensity in the choice of the task. We asked the question in the context of citizen science, wherein the interested public engages in scientific activities, such as data collection and analysis^25^. We developed a computer platform where users perform environmental monitoring activities collaboratively with a computer-programmed virtual partner, toward assembling an image repository of a notoriously polluted canal in the U.S. (the Gowanus Canal, Brooklyn, NY). From their choice of the task during the activities, we investigated the plasticity in leadership and followership in task cooperation. From their movement trajectories, we quantified responsiveness to the partner’s movement using transfer entropy to identify leadership and followership in movement coordination.

## Results

**Figure 1:**
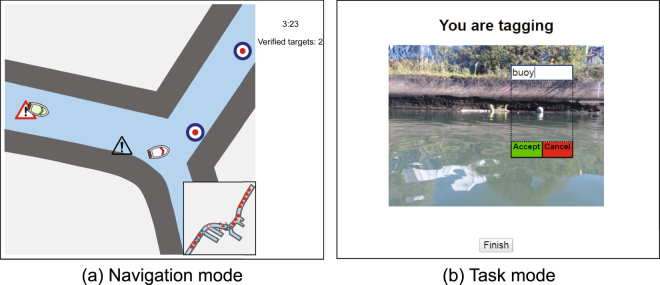
Computer platform for the experiment. On the navigation mode (**a**), users move a boat toward a task location, which is displayed with different symbols depending on the states (untagged, tagged by a user, and tagged by a virtual partner). A mini map is presented at the bottom right to show the positions of their virtual partner and task locations. On the task mode (**b**), users either create image tags or validate tags created by their virtual partner at each task location.

In the computer platform, participants started simultaneously with their virtual partner at the same endpoint of an unbranched path on the canal. Both were free to move forward and backward individually along the canal toward one of the equidistantly spaced locations (Fig. 1a). At each location, participants were able to view a picture taken in the canal^26^ and either create image tags by typing the description or validate the tags created by their virtual partner by approving or correcting them (Fig. 1b). We asked participants to maximize the number of validated images (i.e., tagging followed by validation) collaboratively with their virtual partner. Because both parties were not allowed to validate the tags created by their own, they needed to cooperate in their choice of the task without free-riding to achieve the goal. In this setting, the dichotomous tasks coincided with social roles of leader and follower in task cooperation: *tagging* can be considered as *leading*, and *validation* as *following*.

Participants were randomly paired with a virtual partner with a contrasting propensity to lead or follow in the choice of the task. One virtual partner was programmed to lead more, with a probability of tagging over validation 0.7, and the other was programmed to follow more, with a probability of tagging 0.1, when both tasks were available (corresponding to 90% and 10% quantiles of the preference in our preliminary experiment, see Supplementary Materials). In case only one task was available, the virtual partner automatically selected that one. Movement of the virtual partner was calibrated to mimic that of humans from the preliminary experiments (see Supplementary Materials). Participants were informed that the virtual partner was another participant at a remote location.

### Leader–follower roles in cooperation

**Figure 2:**
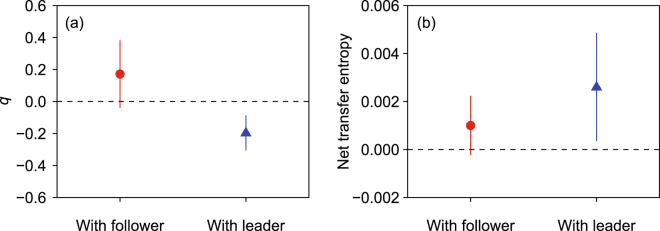
Extent of leader–follower roles in (**a**) task cooperation and (**b**) movement coordination. (**a**) The score *q* takes −1 when an individual always follows their virtual partner, 1 when an individual is always followed by their virtual partner, and 0 when there is no leader–follower relationship between the two in the choice of the task. (**b**) Positive net transfer entropy from a virtual partner to an individual indicates movement of the virtual partner predict that of an individual, and negative net transfer entropy indicates the opposite. Points and vertical lines indicate means and 95% confidence intervals, respectively.

Individuals exhibited their social roles differently in response to their virtual partner’s propensity to lead or follow in the choice of the task when performing a collective activity in pairs (Fig. 2a). When paired with a virtual partner that had a stronger propensity to lead than to follow, individuals were more likely to take a follower role in the choice of the task (*q* = −0.196 ± 0.246, mean ± standard deviation, *t* = −3.732, *df* = 21, *p* = 0.001). By contrast, when paired with a virtual partner that had a stronger propensity to follow, individuals displayed only a weak tendency to lead in choice of the task (*q* = 0.172 ± 0.424, *t* = 1.719, *df* = 17, *p* = 0.104). Individuals tagged a similar number of images regardless of the propensity of their partner in the choice of the task (8.0 ± 3.2 vs. 8.8 ± 4.7, ${\chi }_{1}^{2}$ = 0.804, *p* = 0.370, Poisson GLM).

Because tagging took longer than validation (18.5 ± 16.6 s vs. 10.1 ± 8.3 s, ${\chi }_{1}^{2}$ = 238.39, *p* < 0.001, gamma GLMM with individual as a random effect), the virtual follower occasionally encountered situations where there was no choice other than to tag after tentatively completing all validation tasks. Consequently, it validated less than programmed (0.443 ± 0.237 for validation, one-sample *t*-test against 0.9, *t* = −8.180, *df* = 17, *p* < 0.001).

When compared between the first and second halves of the trials, there was no difference in the extent of leader–follower roles in cooperation in pairs with a virtual leader (paired *t*-test, *t* = 0.128, *df* = 21, *p* = 0.899) or those with a virtual follower (*t* = 0.562, *df* = 17, *p* = 0.582).

### Leader–follower roles in coordination

**Figure 3:**
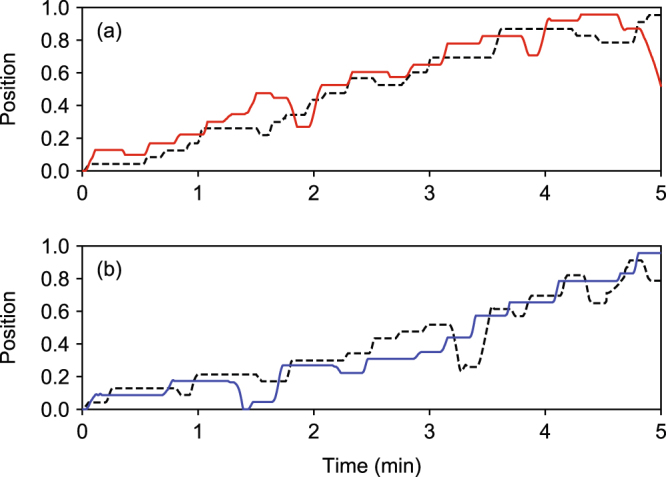
Examples of boat movements by humans and virtual partners: (**a**) when a virtual partner had a strong propensity to follow in the choice of the task, and (**b**) when a virtual partner had a strong propensity to lead in the choice of the task. Solid lines represent the trajectory of the individual, and dashed lines represent that of the virtual partner. The horizontal segments of the trajectories indicate when the human and virtual partner were performing either the tagging or validation tasks.

Differences in taking leader and follower roles in the choice of the task were accompanied by dynamic adaptation in moving through the canal. Individuals responded to the virtual partner’s movements by adjusting their own movements through the canal (Fig. 3). When interacting with a virtual leader, individuals tended to lag in their movements, while they were prone to anticipate the movements of a virtual follower.

The analysis of transfer entropy showed contrasting patterns of leader–follower roles during the movement between the task locations in response to a virtual partner’s propensity to be a leader or a follower in the choice of the task (Fig. 2b). Transfer entropy from an individual to a virtual partner was similar between pairs with a virtual leader and a virtual follower (0.012 ± 0.006 vs. 0.010 ± 0.004, Welch’s *t*-test, *t* = 1.662, *df* = 37.157, *p* = 0.105). By contrast, transfer entropy from a virtual partner to an individual was greater in pairs with a virtual leader, compared to those with a virtual follower (0.015 ± 0.008 vs. 0.011 ± 0.004, Welch’s *t*-test, *t* = 2.111, *df* = 33.943, *p* = 0.042).

Consequently, we found a positive net transfer entropy (*NetTE*) from a virtual partner to an individual in pairs with a virtual leader (*NetTE* = 0.0026 ± 0.0051, *t* = 2.403, *df* = 21, *p* = 0.026), indicating that the movement of an individual was predicted by that of the virtual partner. By contrast, in pairs with a virtual follower, neither movement of the individual nor virtual partner was successful in predicting the movement of the other (*NetTE* = 0.001 ± 0.002, *t* = 1.702, *df* = 17, *p* = 0.107). The temporal resolution that provided the strongest signal was 0.18 s (*τ* = 11) for pairs with a virtual leader and 0.17 s (*τ* = 10) for pairs with a virtual follower, which corresponds to the range of reaction time to visual stimuli in other studies^27,28^.

Toward validating the use of transfer entropy to quantify social responsiveness, we found no net transfer entropy between the two virtual agents with the contrasting propensities to lead and follow working together (*NetTE* = −0.001 ± 0.002, *z* = −0.438, *p* = 0.662, 1,000 simulations). In this case, both moved between task locations only based on the task availability, without responding to the movement of the other in real time.

When compared between the first and second halves of the trials, there was no difference in net transfer entropy in pairs with a virtual leader (paired *t*-test, *t* = 1.045, *df* = 21, *p* = 0.308) or those with a virtual follower (*t* = 0.485, *df* = 17, *p* = 0.634).

### Team performance

A stronger propensity of a virtual partner to lead resulted in a poorer team performance. The number of locations visited by both was smaller in pairs with a virtual leader than in those with a virtual follower (9.1 ± 4.2 vs. 12.4 ± 3.1, ${\chi }_{1}^{2}$ = 10.437, *p* = 0.001, Poisson GLM). However, individual efforts, estimated as the total number of locations visited by an individual, were similar regardless of the virtual partner’s propensity in the choice of the task (13.9 ± 4.1 vs. 13.4 ± 3.6, ${\chi }_{1}^{2}$ = 0.156, *p* = 0.693, Poisson GLM). Consequently, individuals contributed less to group performance in pairs with a virtual leader compared to those with a virtual follower, when it was evaluated as a proportion of the number of locations visited by both over the number of locations visited by an individual (0.65 ± 0.25 vs. 0.93 ± 0.06, ${\chi }_{1}^{2}$ = 63.041, *p* < 0.001, binomial GLM).

**Figure 4:**
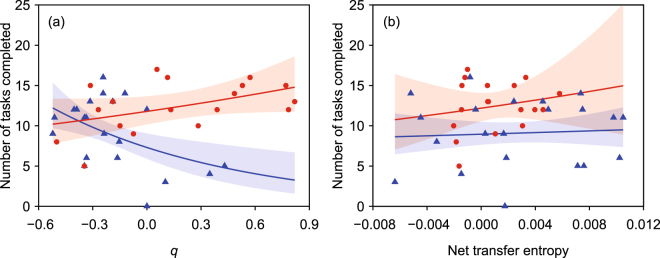
The number of tasks completed as a pair in relation to leadership in (**a**) task cooperation and (**b**) movement coordination. Circles indicate individuals paired with a virtual follower, and triangles indicate individuals paired with a virtual leader. Shaded areas represent 95% confidence bands of the estimates. There was a significant interaction between *q* and the type of virtual partner, while there was no interaction between net transfer entropy and the type of virtual partner.

Team performance was also explained by the extent of leadership and followership expressed by individuals (Fig. 4). In task cooperation, a significant interaction was found between *q* and the type of virtual partner (${\chi }_{1}^{2}$ = 12.266, *p* < 0.001, Poisson GLM). In movement coordination, by contrast, there was no interaction between *NetTE* and the type of virtual partner (${\chi }_{1}^{2}$ = 0.205, *p* = 0.651, Poisson GLM). When the interaction term was removed from the model, team performance was explained by the type of virtual partner (${\chi }_{1}^{2}$ = 10.899, *p* < 0.001), but not by *NetTE* (${\chi }_{1}^{2}$ = 0.462, *p* = 0.497).

## Discussion

This study demonstrates adaptive plasticity in taking leader–follower roles in task cooperation, where one takes a leader role when a partner is prone to follow, but also takes a follower role when the partner occasionally leads in the choice of the task. The partner’s propensity in the choice of the task was reflected to the social roles of leader and follower in movement coordination. When paired with a partner with a strong propensity to lead in the choice of the task, individuals were more responsive to their partner’s movement. By contrast, individuals did not exert strong leadership in movement coordination when paired with one with a strong propensity to follow in the choice of the task.

A review on human leadership literature identifies over 90 dimensions that could define leadership^29^. Here, focusing on the aspects of leadership in task cooperation and movement coordination, we found a correspondence between taking leader–follower roles with respect to choosing the task of tagging and validation and moving the boat along the canal on the screen. It is unlikely that the correspondence is an artifact of the experimental design, considering that cooperation in the choice of the task does not necessitate coordination in movement, and vice versa, in our experiment. In effect, no net transfer entropy was found when the collective activity was performed by two virtual agents, which move only based on the task availability and their preference, without social responsiveness. Therefore, the correspondence indicates that a stronger leadership of a virtual partner in the choice of the task elicited a stronger followership in both task cooperation and movement coordination.

As expected, individuals adaptively adjusted the social roles of leader and follower in task cooperation in response to their partner’s propensity. However, the extent of the response to their virtual partner depended on their partner’s propensity. We offer two alternative explanations for the registered asymmetry, whereby strong human followership in task cooperation emerged during the interactions with a virtual leader and only a weak leadership was found in humans paired with a virtual follower. First, the weakness in human leadership in task cooperation when paired with a virtual follower could be related to a difference between the virtual partner’s propensity to follow in the choice of the task and the executed action of doing so. Because tagging took longer than validation, the virtual follower occasionally encountered situations where there was no choice other than to tag after tentatively completing all validation tasks. Consequently, it validated less than programmed. Second, the lack of a significant human leadership may be explained by a stronger preference of humans for the validation task when they were free to choose without any social information (see Supplementary Materials), which may have counteracted the manifestation of leadership.

The same asymmetry was found in the extent of plasticity in the social roles of leader and follower in movement coordination. Our results on transfer entropy suggest that individuals followed the virtual partner’s movement when paired with a partner that had a stronger propensity to lead in the choice of the task. By contrast, we did not find distinct leader–follower roles in movement coordination when the virtual partner had a stronger propensity to follow in the choice of the task. This indicates that when paired with a virtual follower, individuals were more likely to ignore information on partner’s movement and move only based on the task availability, as seen in the two virtual agents working on the collective activity. The strong human followership to the virtual leader in coordinated movement could have arisen from uncertainty in the expectation of the partner’s choice of the tasks. Although virtual partners had a certain probability to choose one action over the other in the task, the probability were unknown to the participants. Thus, when a partner is more leading in the choice of the task, humans may not be able to learn and predict the partner’s choice. Indeed, we found no difference in net transfer entropy between the first and second halves of the experiment, indicating that individuals continuously followed their virtual leader in movement, possibly to gain information on their partner’s future choice of the task.

Interestingly, a stronger propensity of a virtual partner to lead in the choice of the task resulted in a poorer team performance. The difference in team performance may have arisen from how the virtual partner responded to the leading actions of individuals. Because validation was quicker than tagging, individuals had time to visit one of the locations to tag while their partner was still tagging. However, the leading actions of individuals did not contribute to the team performance in pairs with a virtual leader, because the virtual leader followed less in the choice of the task as it was programmed to do so. Thus, the performance loss was likely due to the increased number of actions that were not followed by the virtual leader. In line with our results, a study on team performance found that task-oriented leadership led to higher group efficacy, whereas relationship-oriented leadership led to greater cohesions among group members^30^. Our results indicate that, when people try to collaborate toward a shared goal with limited resources (time in our experiment), Machiavellian leaders may hamper team performance, but adaptive plasticity could avert the damage to some extent.

In addition to the partner’s propensity, individual differences in responding to their virtual partner explained team performance. Stronger leadership in task cooperation resulted in higher team performance when individuals were paired with a virtual follower, whereas it led to poorer team performance when they were paired with a virtual leader. However, the extent of leadership in movement coordination did not influence team performance. These results satisfy our expectations, in which taking the opposite roles of their partner in task cooperation is adaptive to achieve their objective of maximizing the number of tasks completed as a team. The observed deviation from the adaptive behavioral response may be attributed to individual differences, such as task preference, motivation, and social responsiveness. If both members are allowed to change their behavior in response to each other, positive social feedback may facilitate behavioral differences between the interacting members, resulting in stronger behavioral consistency in leadership and followership^31^, along with higher group synchrony^32^ and higher group performance^33^.

In summary, our results demonstrate that humans adaptively adjust their social behavior in response to the social environment when collaborating toward a shared goal. By eliminating social feedback between interacting agents through a computer-programmed virtual partner, and quantifying social responsiveness in movement coordination through transfer entropy, this study advances our understanding of behavioral plasticity in cooperation and coordination. Humans expressed adaptive plasticity in the social roles of leader and follower in both task cooperation and movement coordination in response to their partner’s propensity, and the extent of plasticity depended on their partner’s propensity in the choice of the task. Our findings suggest the possibility of eliciting behavioral plasticity through design interventions, toward an enhanced group performance when people collaborate in virtual settings. Although we focused on behavioral plasticity in response to the social environment, humans could also exhibit behavioral plasticity in response to temporal changes in the social environment. Further study is needed to understand behavioral resilience after an initial adaptation, by manipulating the propensity of social partners during the experiment.

## Methods

The Brooklyn Atlantis citizen science project was launched to monitor the environmental status of the Gowanus Canal (Brooklyn, NY, USA), one of the most polluted bodies of water in the country, with help of robotic technology^26,34^. A robot was designed to navigate in the canal and automatically collect images with a camera on board. In addition, it was equipped with sensors to collect data on water quality, such as temperature, dissolved oxygen level, and pH. The data were sent to our server in real time via GPS and made accessible to the public on the dedicated website.

### Experimental platform

The computer platform was designed in such a way that users could maneuver a boat along the canal towards predetermined locations and perform environmental monitoring activities in collaboration with a virtual partner. A computer screen displays a zoomed map of the Gowanus Canal, the boats of the user and the virtual partner, and icons of the task locations (Fig. 1). The whole map of the canal is also shown on the bottom-right corner of the screen, so that the users are always able to perceive the positions of the virtual partner and the task locations.

The users can maneuver the boat northeast and southwest along the predetermined path in the canal, by pressing the upper-right button of the game pad with the right index finger and the upper-left button with the left index finger, respectively. One button press feeds an instantaneous acceleration of 0.01 unit/frame to a desired direction on the path, where a total length of the path is set to 1 unit and the location of the boat is updated at 60 fps. The speed of the boat decays as *v*_*t* + Δ*t*_ = *v*_*t*_ *e* ^−*λ*Δ*t*^, where *v*_*t*_ and *v*_*t* + Δ*t*_ are the speeds at time *t* and *t* + Δ*t*, and *λ* is an exponential decay constant. We set *λ* = 0.01/frame. Consequently, the users need to repeatedly press the button to achieve a long-distance movement.

When the users reach one of the locations, they can choose to perform an environmental monitoring activity by pressing a dedicated button of the game pad with the right thumb. The activity consists of two tasks: tagging and validation. In the tagging task, the computer screen randomly displays one of the images previously taken by our aquatic robot^26^ on the canal. The users can click on any noticeable objects in the image using a mouse and type the description using a keyboard (e.g., tree, canoe, and factory). The tag is then displayed on the image. They can create as many tags as they want on the same image. In the validation task, the users are presented with one of the images, but this time, with a tag. They can validate the description by clicking the tag and selecting either ‘Correct’ or ‘Incorrect’. When selecting ‘Incorrect’, they can update the description using a keyboard. After completing the activity, the users are redirected to boat navigation by pressing ‘Finish’ displayed on the bottom of the image. The users perform the tagging task when they visit there before the partner and the validation task when they visit there after the partner. Initially, there are 20 locations evenly spaced along the canal. The location icon on the map changes in response to the status of the location (i.e., untagged, tagged by the user, or tagged by the virtual partner). When the location is visited by both the user and the partner, the icon disappears from the map.

The virtual partner is programmed to find the two nearest locations: one is untagged, and the other is tagged by the user. When both exist, it selects one with a certain probability and moves towards it. From a preliminary experiment (*N* = 24), we set a probability of 0.7 to select a tagging task over a validation task for the virtual partner with a stronger propensity to lead in the choice of the task (‘virtual leader’), and 0.1 for that with a stronger propensity to follow in the choice of the task (‘virtual follower’). The probabilities were chosen from the 10% and 90% quantiles of the preference measured as the number of each task performed in the preliminary experiment. In case there is only one option available, the virtual partner automatically chooses it. When the target location is visited by the user while still moving toward it, the virtual partner instantly updates the target location with the same decision rule. The movement traits of the virtual partner were programmed to mimic human behavior from another preliminary experiment (*N* = 10). Upon arrival at the location, it spends a predetermined time from a pilot data fitted to a gamma distribution (13.5 s for tagging and 10.0 s for validation on average), and resumes to move to the next location. The detailed movement traits of the virtual partner are found in Supplementary Materials.

### Experimental procedure

We set up a research stand on an outdoor promenade in front of a large supermarket by the Gowanus Canal. The location was selected to attract local people who were likely to be concerned about the pollution problem of the canal. The research stand was appealed to passers-by with a table cover with a university logo and the aquatic robot displayed on the table. We explained the project to the people who approached the stand and asked for the participation in the experiment.

Upon voluntary consent, people participated in the experiment using a laptop on a table, while sitting on a chair, at the research stand. First, the experimenter explained how to create and validate tags on images, and the participants practiced the tagging and validation for 2 min. Next, the participants were introduced to a hands-on practice of boat navigation and location selection using a game pad, during which they were explained the meanings of location icons on the map. Finally, the participants proceeded to the main session in which they controlled a boat along the map of the canal and performed the tagging and validation tasks in collaboration with their partner for 5 min.

Before the main session, the experimenter explained to the participants that the goal of the exercise was to maximize the number of validated locations as a team toward assembling an image repository, and that they could not validate the tags created by their own. Each participant was randomly paired with one of the two virtual partners that has a contrasting propensity to lead or follow. The pair started simultaneously at a position 0 locating at a bottom-left end of the canal. The current number of the validated locations and the remaining time were displayed at the top-right corner of the screen during the main session. The session was terminated before 5 min when there was no location left for one of the pair to tag or validate. The experimenter was sitting next to the participant to assist them during the whole session, and they were allowed to ask questions at any time. The participants were told that the virtual partner was another participant at a remote location. After the experiment, the participants were debriefed.

In total, 40 participants engaged in the experiment, with 22 participants paired with a partner that had a stronger propensity to tag, and 18 participants with a partner that had a stronger propensity to validate. All participants were over 18 years old, and their personal information was not collected. All participants attested their voluntary participation by signing an informed consent form before the experiment. All experiments were approved by the institutional review board of New York University (IRB-FY2016-184) and performed in accordance with relevant guidelines and regulations.

### Quantifying leader–follower roles in cooperation

To investigate how individuals changed the social roles of leader and follower in the choice of the task in response to the partners’ propensity to lead or follow, we applied a modified version of event synchronization analysis^35^. Specifically, we checked the order of arrivals at each location when the tagging event was followed by the validation event, while taking into account the total number of tagging and validation events performed by both in the pair. The ordered event synchronization score was calculated as

1 $$q=\frac{{n}_{{y}^{i}|{x}^{i}}-{n}_{{x}^{i}|{y}^{i}}}{\sqrt{{n}_{{x}^{i}}{n}_{{y}^{i}}}},$$

where *n*_*y*_^*i*^_|*x*_^*i*^ is the number of events an individual *i* (*i* = 1, …, 40) visited the task locations and followed by their partner, *n*_*x*_^*i*^_|*y*_^*i*^ is the number of events an individual visited the task locations following their partner, *n*_*x*_^*i*^ is the total number of task locations visited by an individual *i*, and *n*_*y*_^*i*^ is the total number of task locations visited by their partner. The score *q* ranges from −1 to 1, with 1 indicating that an individual is always followed by their partner, −1 indicating that an individual always follows their partner, and 0 indicating that there is no leader–follower relationship between the two in the choice of the task.

To investigate whether individuals changed leadership and followership in task cooperation over time, we divided the data into two composed of the first and second halves of the period and quantified *q* in the same way. The changes in *q* were tested using a paired *t*-test for individuals paired with a virtual leader and a virtual follower, respectively.

### Quantifying leader–follower roles in coordination

To investigate how individuals responded to the movement of their partners, we used the notion of transfer entropy. Transfer entropy measures the reduction in the level of uncertainty (i.e., entropy) in predicting the future state of a system, given the current state of the other system. In coordinated movement, transfer entropy can be applied to measure social responsiveness to others by quantifying the reduction in uncertainty in predicting one’s future movement given the current movement of the other. That is, it can identify leader–follower relationship between the two in coordinated movement.

To overcome the strong nonlinear behavior and the sensitivity to outliers in the dynamics of the time series, we embedded the time series in an *m*-dimensional space and constructed symbols^36^ as follows. Let *z* be a time series of a balanced ternary system that takes one of the three discrete variables at each time step. Here, *z*_*t*_^*τ*,*m*^ = (*z*_*t*_, *z*_*t* + *τ*_, …, *z*_*t* + *τ*(*m* − 1)_) is a symbol composed of the *m*-history of *z* starting at *t* with an interval of *τ*. The amount of information of every *m*-history forms a random variable whose expected value, on average, is the Shannon entropy of

2 $$H({z}_{t}^{\tau ,m})=-\sum _{\sigma \in {\rm{\Gamma }}}{\rm{\Pr }}({z}_{t}^{\tau ,m}=\sigma )\mathrm{log}\,{\rm{\Pr }}({z}_{t}^{\tau ,m}=\sigma ),$$

where Γ denotes each of the 3^*m*^ possible realizations of the symbols. The summation is over all the possible realizations of the *m*-histories and the base of the logarithm is chosen to be equal to 2, following standard practice in information theory. For two *m*-histories *z*_*t*_^*τ*,*m*^ and *w*_*t*_^*τ*,*m*^, the joint entropy and the conditional entropy are defined respectively as

3 $$H({z}_{t}^{\tau ,m},{w}_{t}^{\tau ,m})=-\sum _{(\sigma ,\pi )\in {\rm{\Gamma }}\times {\rm{\Gamma }}}{\rm{\Pr }}({z}_{t}^{\tau ,m}=\sigma ,{w}_{t}^{\tau ,m}=\pi )\mathrm{log}\,{\rm{\Pr }}({z}_{t}^{\tau ,m}=\sigma ,{w}_{t}^{\tau ,m}=\pi ),$$

4 $$H({z}_{t}^{\tau ,m}|{w}_{t}^{\tau ,m})=-\sum _{(\sigma ,\pi )\in {\rm{\Gamma }}\times {\rm{\Gamma }}}{\rm{\Pr }}({z}_{t}^{\tau ,m}=\sigma ,{w}_{t}^{\tau ,m}=\pi )\mathrm{log}\,{\rm{\Pr }}({z}_{t}^{\tau ,m}=\sigma |{w}_{t}^{\tau ,m}=\pi \mathrm{).}$$

Information transfer from *w* to *z* is measured by the symbolic transfer entropy

5 $$T{E}_{w\to z}^{\tau ,m}=H({z}_{t+1}^{\tau ,m}|{z}_{t}^{\tau ,m})-H({z}_{t+1}^{\tau ,m}|{z}_{t}^{\tau ,m},{w}_{t}^{\tau ,m}\mathrm{).}$$

This quantity can be viewed as the reduction in uncertainty in predicting the future state of *z* (more precisely, the future state of a symbol *z* ^*τ*,*m*^), by knowing the current states of *w* and *z*. Thus, transfer entropy offers a measure of predictability between the time series^15^. Large transfer entropy from *w* to *z* indicates that the future state of *z* is predicted by the current state of *w*, whereas small transfer entropy indicates that the future state of *z* is independent of the current state of *w*.

Transfer entropy from *w* to *z* is generally different from that in the opposite direction. Net transfer entropy is measured as

6 $$NetT{E}_{w\to z}^{\tau ,m}=T{E}_{w\to z}^{\tau ,m}-T{E}_{z\to w}^{\tau ,m}\mathrm{.}$$

A positive net transfer entropy indicates that *w* predicts *z*, a negative value indicates the opposite, and zero indicates a lack of a relationship between the two. We scored transfer entropy from the movements of boats by the individual and virtual partner in each trial. To investigate leader–follower relationship in coordinated movement, we computed transfer entropy in each pair’s movement. First, we converted the movement of an individual *i* (*i* = 1, …, 40) to a ternary system *x* ^*i*^ by assigning −1 when they moved to the left, 0 when they stayed, and 1 when they moved to the right at each time step (60 fps). Similarly, the movement of their virtual partner was converted to a ternary system *y* ^*i*^. Second, these time series were embedded in *m*-histories to construct time series of the symbols, *x* ^*i*,*τ*,*m*^ and *y* ^*i*,*τ*,*m*^. Finally, to identify the dominant direction of information transfer, we measured net transfer entropy *NetTE*_*y*_^*i*^_→ *x*_^*iτ*,*m*^, with *m* = 3. Positive net transfer entropy indicates that the current movement of a virtual partner exerted a stronger influence on the future movement of an individual than the opposite direction. We checked the net transfer entropy for *τ* = 2, …, 60 and selected *τ* that maximizes the absolute value of the effect size in each condition.

To investigate whether individuals changed social responsiveness to the partner’s movement over time, we divided the movement trajectories into two composed of the first and second halves of the period and quantified net transfer entropy in the same way. The changes in the net transfer entropy were tested using a paired *t*-test for individuals paired with a virtual leader and a virtual follower, respectively.

To validate the use of net transfer entropy to quantify social responsiveness in movement, we simulated the movements of two virtual partners with the contrasting propensities to lead and follow working together. Because the virtual partners were programmed to move only based on the task availability and preference, we expected no net transfer entropy between these pairs regardless of the strong leader–follower relationship in task cooperation. From 1,000 pairs of simulated movement trajectories, we tested the net transfer entropy against zero using a *z*-test.

### Data availability

The data analyzed during the current study are available in Supplementary Materials.

## Electronic supplementary material

Supplementary Information

Dataset 1
