# Supplementary material for: Plasticity in leader–follower roles in human teams
Source: Sci Rep. 2017 Nov 6;7:14562. doi: 10.1038/s41598-017-14851-6 (PMC5673975; doi:10.1038/s41598-017-14851-6)
Supplement: Supplementary file 1 — Supplementary Information [file 41598_2017_14851_MOESM1_ESM.pdf]

Plasticity in leader–follower roles in human teams

Shinnosuke Nakayama[1], Manuel Ruiz Marín[2], Maximo Camacho[3], and Maurizio Porfiri[1,*]

[1]Department of Mechanical and Aerospace Engineering, New York University Tandon School of Engineering, 6 Metrotech Center, Brooklyn, NY, 11201, USA
[2]Department of Quantitative and Informatic Methods, Technical University of Cartagena, Plaza Cronista Isidoro Valverde, 30202, Cartagena, Spain
[3]Department of Quantitative Methods for Economics and Business, University of Murcia, Campus de Espinardo, 30100, Spain
[*]mporfiri@nyu.edu

Supplementary Information

Movement rules of the virtual boat controlled by the user

A user can move the boat northeast and southwest along the predetermined path in the canal, by pressing the upper right button of the game pad with the right index finger and the upper left button with the left index finger, respectively. One button press feeds an instantaneous acceleration of 0.01 unit/frame to a desired direction on the path, where the total length of the path is set to 1 unit and the location of the boat is updated at 60 fps. The speed of the boat decays as $v_{t+\Delta t} = v_t e^{-\lambda \Delta t}$, where v_t and $v_{t+\Delta t}$ are the speeds at time t and $t+\Delta t$, and λ is an exponential decay constant. We set $\lambda = 0.01$/frame. Consequently, a user needs to repeatedly press the button to achieve a long-distance movement. The maximum speed of the boat is set at 0.015/frame to be able to enter a target location when the boat passes over it.

Movement rules of the virtual partner

The trajectory of the boat maneuvered by a virtual partner was programmed to closely mimic the trajectories generated by humans. We performed a preliminary experiment with voluntary university students ($N = 10$) to identify the movement traits of the virtual partner. Students were instructed to navigate the boat to a target displayed on the map and press a button on the game pad when they reach it. The next target was displayed at a random location in the canal immediately after pressing the button. The process was repeated 20 times for each student.

From these data, we estimated the frequency of pressing the acceleration button and the duration from the appearance of a target to the first press of the acceleration button. After visually examining these data, we found a general pattern of how users pressed the acceleration button. First, there was a delay from the appearance of the new target to a first button press. Then, users pressed the acceleration button at regular intervals during each excursion. There was a clear point at which users changed the patterns of button press, by either decreasing the frequency of button press, completely stopping and coast to the target location, or decelerating by pressing the opposite button. We visually determined the point at which users changed the acceleration patterns, and the intervals of button press were measured between the start delay and the point where users switched the behavior.

The start delay was fitted to a gamma distribution (Figure 1a), and the time intervals of pressing the acceleration button were fitted to a Burr distribution (Figure 1b), both using a maximum likelihood estimation method. The point of behavioral switching was fitted to a linear mixed model, with the total distance to the target as a predictor variable, the remaining distance to the target as a response variable, and user identity as a random effect (Figure 2). Both the predictor variable and response variable were log-transformed for a better fit, and the parameters were estimated using a maximum likelihood estimation method.

At each start, a virtual partner finds the nearest target location for tagging and validation respectively, and decides one at a certain probability. If there is only one option available, it automatically selects it. A virtual partner delays the start for a period randomly drawn from the distribution of the start delay (Figure 1a). Then, it moves to the target location by accelerating at intervals randomly drawn from the distribution of the intervals of a button press (Figure 1b), until it reaches the point of behavioral switch. The point was randomly drawn from the distribution of the switching point (Figure 2), which is a function of the total distance to the target location.

When a virtual partner reaches the switching point, it calculates the hypothetical speed if it stopped accelerating and coasted

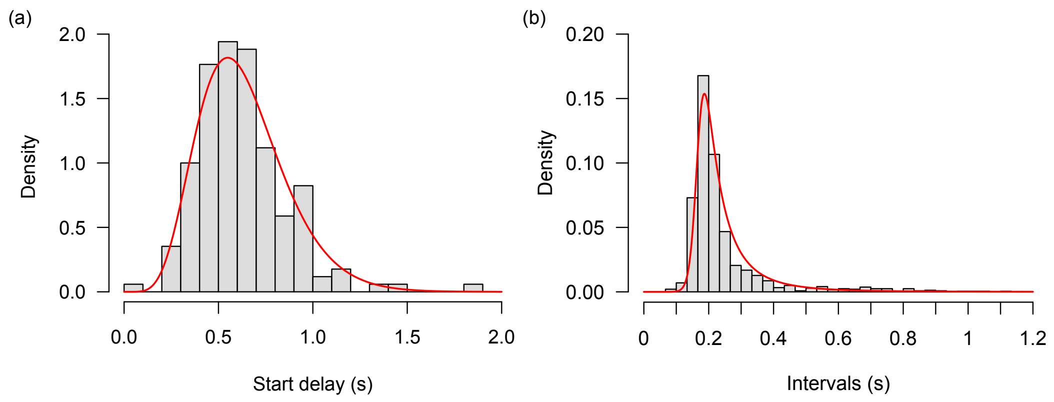

Figure 1. Probability density distribution of (a) the start delay time and (b) the intervals of pressing an acceleration button. The histogram represents the observed data, and the red line is a probability density function after fitting to a gamma distribution.

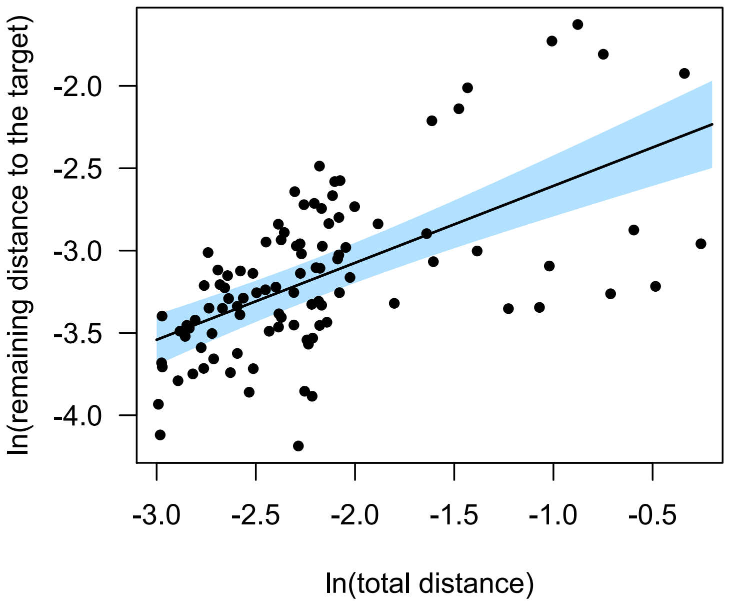

Figure 2. Remaining distance to the target location at which a virtual agent switches the acceleration pattern. Points represent the observed data, and line and shaded area represent the mean estimate and 95% confidence intervals.

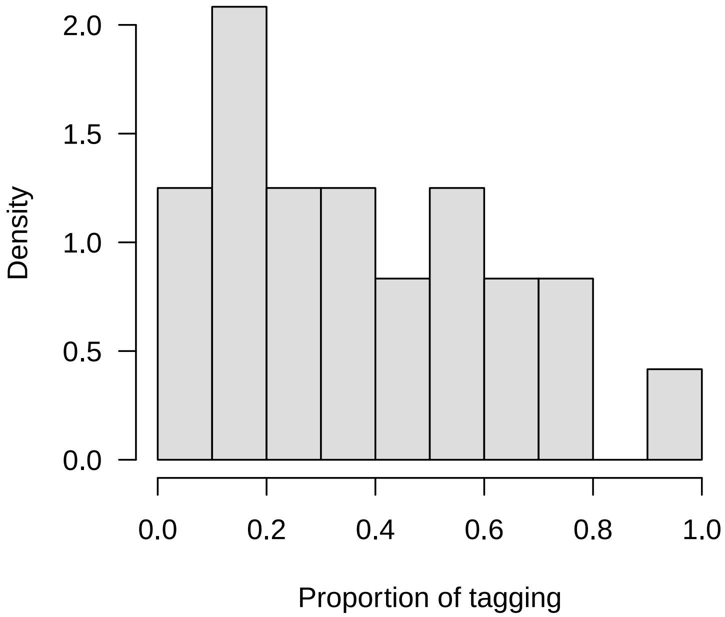

Figure 3. Proportion of tagging over a total number of images processed.

to the target location. If it would reach the target location without further acceleration, it coasts to the target location. If it would not reach the target location, it keeps accelerating but at one-quarter of the regular intervals. When it would overshoot the target location, it decelerates at the regular intervals. A virtual partner adjusts the acceleration patterns in this way until a virtual agent reaches the target location at a speed less than 0.015/frame.

The traits related to the task choice was identified in another preliminary experiment, also conducted on university students. Students ($N = 24$) were instructed to either create tags on the image or validate the tags that were created by the experimenter in advance. They were allowed to switch tasks freely at any time by clicking ‘Switch tasks’, or move to the next image and continue the same task by clicking ‘Next’ displayed on the bottom of the image, for three minutes.

From these data, we estimated the preference and duration of performing the tagging and validation task. The preference in the task choice was estimated as a proportion of the number of images tagged over a total number of images processed by each each subject (Figure 3). From the data, we set a proportion of choosing tagging 0.1 for a virtual partner with a stronger propensity to follow (i.e., validate), and 0.7 for that with a stronger propensity to lead (i.e., tag), which corresponded to the 10% and 90% quantile of the samples, respectively. The duration of tagging and validation was fitted to a gamma distribution, respectively (Figure 4). When a virtual partner stays at the target location, it spends time randomly drawn from the distribution.

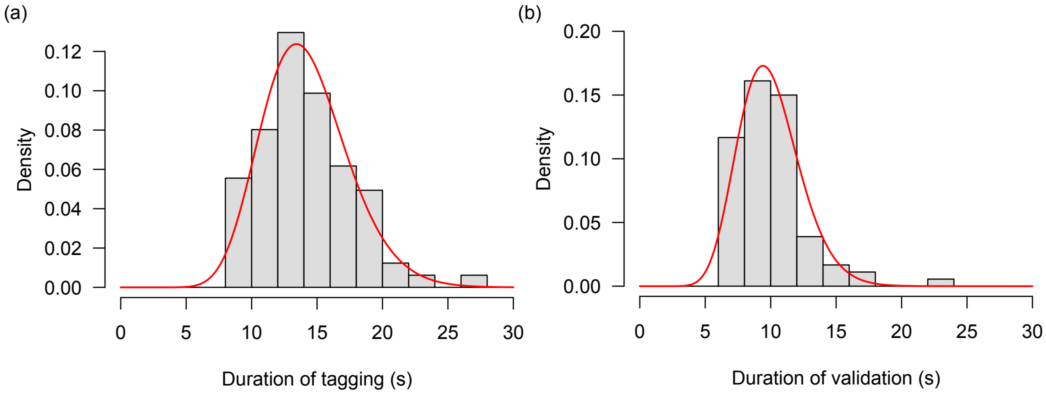

Figure 4. Probability density distribution of the duration of (a) tagging and (b) validation. The histogram represents the observed data, and the red line is a probability density function after fitting to a gamma distribution.
